# Factors That Influence Patient Satisfaction With the Service Quality of Home-Based Teleconsultation During the COVID-19 Pandemic: Cross-Sectional Survey Study

**DOI:** 10.2196/51439

**Published:** 2024-02-16

**Authors:** Guangxia Meng, Carrie McAiney, Ian McKillop, Christopher M Perlman, Shu-Feng Tsao, Helen Chen

**Affiliations:** 1 School of Public Health Sciences University of Walterloo Waterloo, ON Canada

**Keywords:** teleconsultation, secondary stroke prevention, telemedicine, service quality, patient satisfaction

## Abstract

**Background:**

Ontario stroke prevention clinics primarily held in-person visits before the COVID-19 pandemic and then had to shift to a home-based teleconsultation delivery model using telephone or video to provide services during the pandemic. This change may have affected service quality and patient experiences.

**Objective:**

This study seeks to understand patient satisfaction with Ontario stroke prevention clinics’ rapid shift to a home-based teleconsultation delivery model used during the COVID-19 pandemic. The research question explores explanatory factors affecting patient satisfaction.

**Methods:**

Using a cross-sectional service performance model, we surveyed patients who received telephone or video consultations at 2 Ontario stroke prevention clinics in 2021. This survey included closed- and open-ended questions. We used logistic regression and qualitative content analysis to understand factors affecting patient satisfaction with the quality of home-based teleconsultation services.

**Results:**

The overall response rate to the web survey was 37.2% (128/344). The quantitative analysis was based on 110 responses, whereas the qualitative analysis included 97 responses. Logistic regression results revealed that responsiveness (adjusted odds ratio [AOR] 0.034, 95% CI 0.006-0.188; *P*<.001) and empathy (AOR 0.116, 95% CI 0.017-0.800; *P*=.03) were significant factors negatively associated with low satisfaction (scores of 1, 2, or 3 out of 5). The only characteristic positively associated with low satisfaction was when survey consent was provided by the substitute decision maker (AOR 6.592, 95% CI 1.452-29.927; *P*=.02). In the qualitative content analysis, patients with both low and high global satisfaction scores shared the same factors of service dissatisfaction (assurance, reliability, and empathy). The main subcategories associated with dissatisfaction were missing clinical activities, inadequate communication, administrative process issues, and absence of personal connection. Conversely, the high-satisfaction group offered more positive feedback on assurance, reliability, and empathy, as well as on having a competent clinician, appropriate patient selection, and excellent communication and empathy skills.

**Conclusions:**

The insights gained from this study can be considered when designing home-based teleconsultation services to enhance patient experiences in stroke prevention care.

## Introduction

### Secondary Stroke Prevention and Ontario Stroke Prevention Clinics

As of 2019, stroke was the second leading cause of disability worldwide for people aged >50 years, and it was the fourth leading cause of death in Canada from 2017 to 2019 [1,2]. The 36% decline in stroke mortality from 1990 to 2016 can be attributed to better prevention and management of stroke risks [3]. The INTERSTROKE study found that 90% of strokes are preventable owing to modifiable risk factors, including disease-related and behavioral lifestyle factors [4]. Secondary stroke prevention is crucial, as there is up to a 10% risk of recurrent stroke within 90 days of a transient ischemic attack (TIA) or minor stroke [5].

Approximately 80% of patients with minor stroke discharged from the emergency department in Ontario are referred to stroke prevention services [6]. Stroke prevention clinics provide rapid assessments, diagnostic tests, treatments, prevention, and education to reduce the risk of recurrent stroke [7]. Ontario’s 41 stroke prevention clinics are integral to publicly funded health systems [7]. Stroke prevention clinic services are associated with a 25% reduction in mortality [8].

Before the COVID-19 pandemic, stroke prevention care in Ontario was predominantly delivered through in-person consultations. A small percentage of rural and northern Ontario stroke prevention clinics used teleconsultation at local satellite clinics to address access challenges [9]. One stroke prevention clinic conducted a pilot project offering follow-up home video visits from August 2018 to September 2019 [10]. The video consultation produced higher patient satisfaction, was considered safe by physicians, and was shown to be cost-effective in reducing health care costs and patient expenses [10].

In this study, home-based teleconsultation was defined as a synchronous consultation between a clinical service provider and a patient in their home to provide diagnostic or therapeutic advice through telephone or videoconference [11]. Despite a handful of cases in which home-based teleconsultation was used for follow-up care, before the COVID-19 pandemic [10], Ontario stroke prevention clinics had never used home-based teleconsultation to conduct synchronous, interactive, in-home patient visits for new referrals. A survey of >3000 Canadians with stroke, heart disease, or vascular impairment conducted in the spring of 2021 showed that 80% of respondents had had a teleconsultation during the pandemic [12]. The effect of the rapid change from in-person visits to home-based teleconsultation during the COVID-19 pandemic on patients’ experiences was unknown. Patients with stroke are often older adults with multiple chronic conditions [13]. Older adults have lower telehealth service use overall [14]. The impact of service mode change on the older population of stroke prevention clinics needs further exploration.

### Service Quality and Patient Satisfaction

Although the rapid transition to home-based teleconsultation may be a temporary response to the COVID-19 pandemic, it offers a significant opportunity to examine the service quality of home-based teleconsultation in stroke prevention clinics. Delivering safe, high-quality health services is the primary goal of health systems [15]. The literature’s definition of health care service quality is commonly described as including 2 aspects. One views health care service quality as characteristics and features that meet clinicians’ predetermined specifications and standards (such as professional or ethical standards); the other views it as characteristics and features that meet or exceed patients’ needs and expectations [16]. Patients often cannot accurately assess the internal service quality as they lack the medical knowledge to judge [17]; however, patient satisfaction with a medical service is the primary determinant of service quality [18]. Patient satisfaction is essential for and meaningful to delivering high-quality care [19].

Patient satisfaction is generally regarded as patients’ perception of care delivery as well as how their health needs have been addressed [20,21]. Patient satisfaction can be examined using direct and indirect indicators [21]. First, service quality can be measured by directly asking patients to rate their satisfaction with service quality via, for example, a single item with response options ranging from *very dissatisfied* to *very satisfied* [21]. However, the shortfall of single-item measurement is that we can not evaluate or identify a specific aspect of service quality [21]. The alternative approach is to ask patients to rate their experience of different aspects of care, but this indirect measure has the weakness of preemptive assumptions about the determinants of service quality [21]. To obtain an accurate measurement of patient satisfaction, we applied direct and indirect measurements. We asked one question on global satisfaction and applied a theory-guided questionnaire to assess patient satisfaction.

### Service Performance Model

As health care quality is multidimensional, we chose an appropriate service quality model (SERVQUAL) to assess patient satisfaction. Examples of existing health care SERVQUALs include the SERVQUAL [22] and its derivative service performance model (SERVPERF) [23], Total Quality Management [24], Health Quality Model [25], service quality for a public hospital [26], and hospital quality model [27]. The Health Quality Model, service quality for a public hospital, and hospital quality model are derivatives of the SERVQUAL model and assess the care quality of hospital inpatient services. The SERVQUAL and SERVPERF models include 5 dimensions: tangibles, reliability, responsiveness, assurance, and empathy [22]. SERVQUAL tends to measure the difference between one’s expectations and the actual performance of the service [22]. SERVPERF only focuses on the actual performance, and studies have shown that service quality is appropriately modeled using SERVPERF as an antecedent of satisfaction [23,28]. There are 22 service attributes listed within the 5 service dimensions of the SERVPERF model [29]. Textbox 1 presents each dimension and item in detail.

**Dimensions and items of the service performance model (adapted from Zeithaml et al [29]).**

**Tangibles:** **facilities, equipment, and the presence of personnel**

- Up-to-date equipment
- Visually appealing physical facilities
- Neat-appearing employees
- Visually appealing materials associated with the service

**Reliability:** **ability to perform the promised service responsibly and accurately**

- The company keeps its promises to do something by a certain time
- The company shows a sincere interest in solving the customer’s problem
- The company performs the service right the first time
- The company provides its services at the time it promises to do so
- The company insists on error-free records

**Responsiveness:** **willingness to provide help and** **a prompt service to customers**

- Employees of the company tell customers exactly when services will be performed
- Employees of the company provide a prompt service to customers
- Employees of the company are always willing to help customers
- Employees of the company are never too busy to respond to customers

**Assurance:** **the knowledge and courtesy of employees and their ability toinspire trust and confidence**

- Thebehaviorof employees of thecompany instills confidence incustomers
- Customers of the company feel safe in their transactions
- Employees of the company are consistently courteous with customers
- Employees of the company have the knowledge to answer customers’questions

**Empathy:caring and understanding, whicha company provides or offers its customers in terms of its individualized and personalized attention**

- The company gives customers individual attention
- The company has operating hours convenient to all itscustomers
- Employees of the company give customers personal attention
- The company has the customers’ best interests at heart
- The employees of the company understand the specific needs of their customers

### Study Aims and Research Question

Any new service model implementation should usually be well planned to improve user satisfaction; however, home-based teleconsultation at stroke prevention clinics was implemented without the usual planning. The rapid implementation could affect patients’ experiences. As a result, it is vital to evaluate patient satisfaction with the quality of the teleconsultation service they received during the COVID-19 pandemic by assessing satisfaction with various service dimensions to identify aspects of service quality that patients are and are not satisfied with. This study aimed to explore the factors affecting patient satisfaction. The research question was as follows: “what are the patient-identified factors influencing patients’ satisfaction with service quality in stroke prevention clinics’ home-based teleconsultation service?”

## Methods

### Participants and Procedure

We conducted a web-based or telephone survey of patients who had at least one home-based teleconsultation, either the initial or follow-up visit, at the stroke prevention clinics. The study sites were 2 stroke prevention clinics at 2 tertiary hospitals in Ontario, Canada. The study sample consisted of individuals who received at least one home-based teleconsultation at a stroke prevention clinic between January 1, 2021, and November 30, 2021. A convenience sampling technique was used as we invited patients who lived in their homes and self-participated in the stroke prevention clinic home-based teleconsultation service during the COVID-19 pandemic. Our exclusion criteria included (1) patients who lived in a long-term care home or group home and (2) patients with dementia who could not participate in home-based teleconsultations.

To minimize volunteer bias and increase the response rate, we applied various data collection techniques to capture participants with and without internet access. A web survey was used for patients with email addresses, whereas a telephone survey was used for patients without email addresses. The questionnaire was administered between May 17, 2021, and December 10, 2021.

We developed a telephone script for recruitment to explain the research project in lay terms. In total, 2 modified-duty nurses from one site and neurologists from another site who were not part of the research team obtained permission from patients to be contacted by the research team. The list of email addresses or telephone numbers of patients who gave permission was shared with the research team. Participants who chose a web-based survey received an email with a brief cover letter explaining the study’s purpose and their rights as study participants. Informed consent was via the web before accessing the questionnaire, and they were asked to click a box indicating that they agreed to complete the survey (Multimedia Appendix 1). Participants who chose a telephone survey received a mail-in cover letter, a consent form, and a copy of the survey (Multimedia Appendix 2).

### Ethical Considerations

This study was reviewed by the research ethics boards of Southlake Regional Health Center and Mackenzie Health and was considered a continuous quality improvement project; thus, a full research ethics review was not required. This study was also reviewed by the University of Waterloo Office of Research Ethics (ORE 42686) and received ethical clearance.

### Measures

Our study used SERVPERF to design a Likert-scale survey to assess patient satisfaction with home-based teleconsultation service quality in stroke prevention clinics. We acknowledge that patient satisfaction is subjective, with many determinants that may not be related to the SERVPERF model. The literature has indicated that patient satisfaction can be influenced by patient knowledge and expectations; therefore, other factors such as demographics (eg, age, gender, and education), clinical factors (eg, comorbidities, diagnosis, and number of visits), and experiences with teleconsultation can influence patient expectations [20,21]. We also included these factors in our survey. By considering other factors and applying direct and indirect measurements, we attempted to explore patient satisfaction using a holistic approach. The 18-item questionnaire used in our study was developed by referencing the telehealth service quality questionnaire developed by Yin et al [30] (see Multimedia Appendix 3 for a description).

The survey consisted of three components: (1) demographic, clinical, and telemedicine questions; (2) an 18-item Likert scale–based questionnaire measured on a 5-point scale, with 1 for *strongly disagree* and 5 for *strongly agree*; and (3) 6 open-ended follow-up questions (Multimedia Appendix 4). The demographic, clinical, and telemedicine independent variables were selected based on previous evidence from a literature review and clinical significance from a practice point of view [31]. We conducted a pilot study in March 2021 with 10 participants who had home-based teleconsultation from October 2020 to December 2020 and asked 6 additional questions about the survey content (Multimedia Appendix 5). Overall, patients were satisfied with the language and content of the survey, indicated in their feedback.

### Data Collection

The web survey was conducted through a secured, password-protected REDCap (Research Electronic Data Capture; Vanderbilt University) website hosted at the University of Waterloo that supports research data collection [32]. Skype for Business (Skype Technologies) from the University of Waterloo, with recording and transcription functions, was set up for the research assistant to conduct the telephone survey.

### Statistical Analysis

We applied quantitative and qualitative analysis to understand patient satisfaction. A binary outcome variable was defined as (1) a low-satisfaction group if the participants chose *very unsatisfied*, *dissatisfied*, and *neither satisfied nor satisfied* with the overall home-based teleconsultation service quality; and (2) a high-satisfaction group if the participants chose *satisfied* and *very satisfied*. We used SPSS for Windows (version 28.0.1; IBM Corp) for statistical analysis [33]. The Likert-scale questions were converted to numerical values. Using the item means, we generated each SERVPERF dimension score and an overall questionnaire score for each respondent’s survey. There were 10 demographic, 7 clinical, and 6 technical-related independent variables (see Multimedia Appendix 6 for the definitions). Chi-square tests were used to identify the statistical significance between the categorical independent and binary outcome variables. The point biserial correlation was calculated to identify the correlation between a continuous independent variable and the binary outcome variable. To test the internal reliability of our instrument, we calculated the Cronbach α. As we had a large number of independent variables under consideration, a forward selection model was most suitable [34]. We used statistically significant variables correlating to the binary outcome variable in the stepwise binary logistic regression model, with *P*<.05 considered significant.

We used NVivo (QSR International), a software developed to organize and support the analysis of qualitative data. GM coded the entire data set, and ST independently coded 10 random samples. The results were compared and reached an initial 87.1% agreement. Discrepancies were discussed, and conflicts were resolved after further clarification of the definition of the codes. We applied direct content analysis to understand the service quality of the teleconsultation under study [35]. We used the 5 service dimensions and their operational definitions as the initial coding categories [36]. Next, we read each transcript and identified and categorized all the text that appeared to represent the operational definition of the code [35]. Text not categorized using the initial coding scheme would be considered for a new code. We summarized the categories of the entire data set and then divided them into low- and high-satisfaction groups to explore positive and negative patient perceptions. We compared the differences between the 2 groups that could explain the quantitative analysis results [37].

## Results

### Participant Characteristics

**Figure 1:**
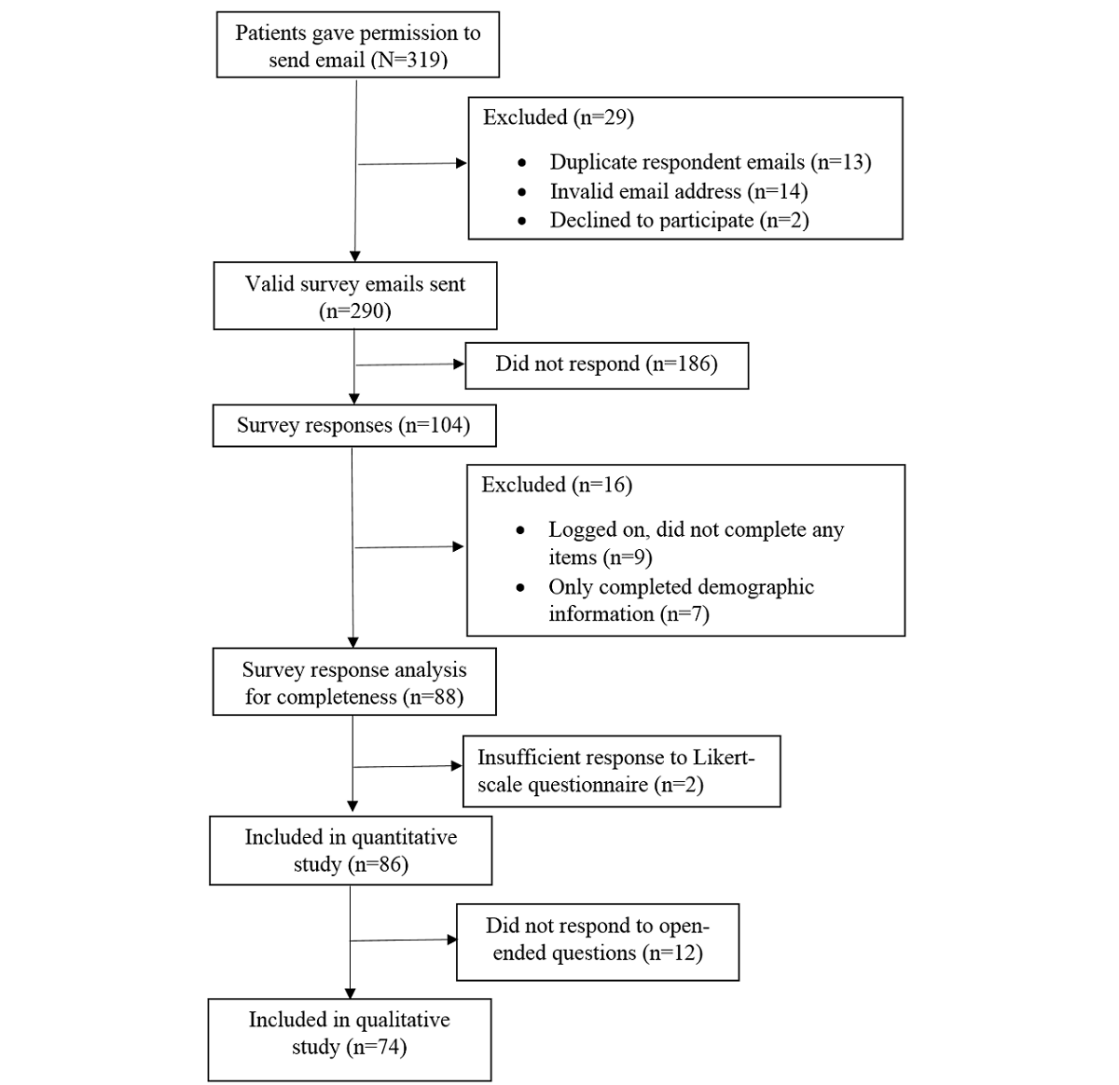
Sample flowchart for the web-based survey.

**Figure 2:**
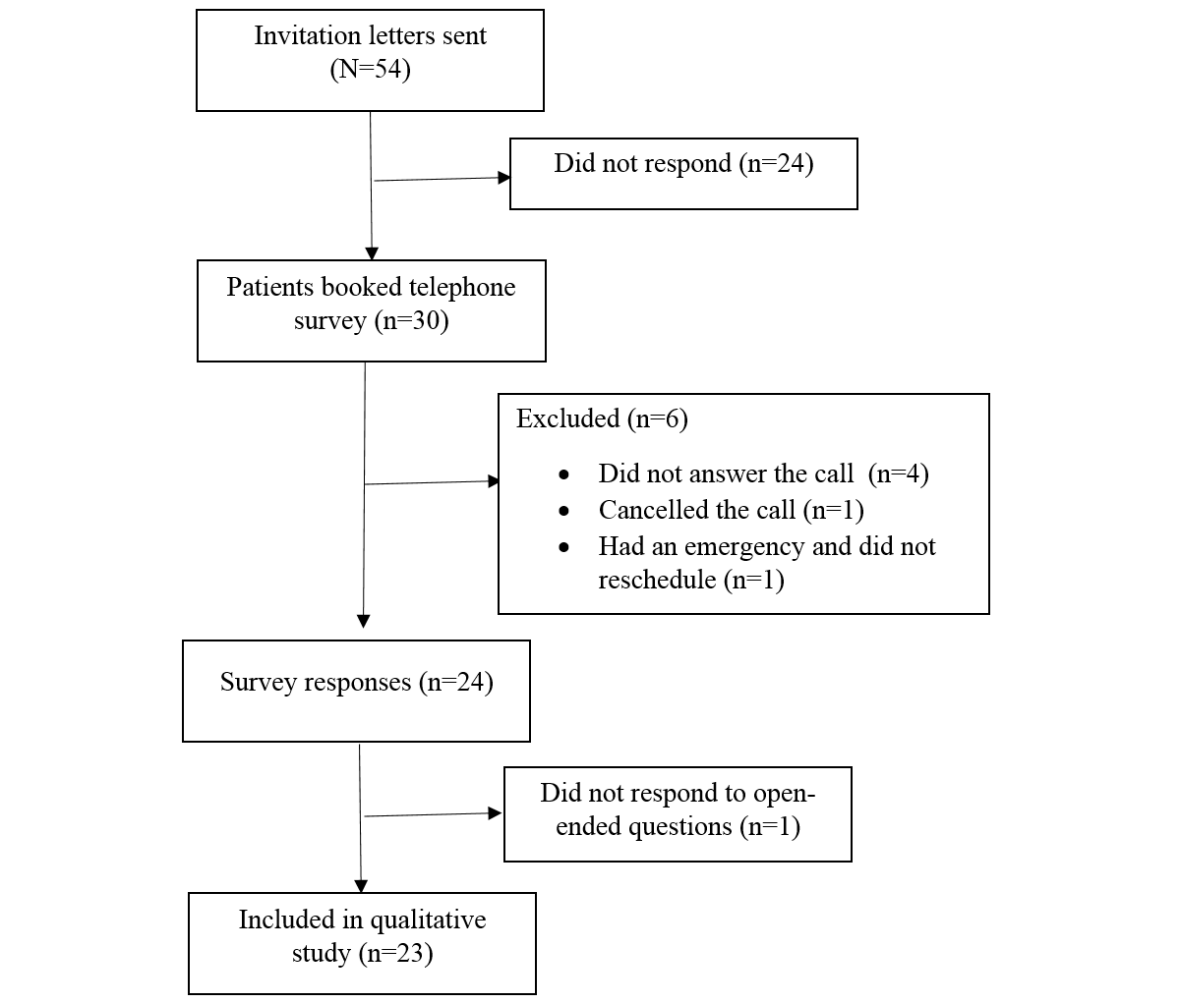
Sample flowchart for the telephone survey.

The response rate was 35.9% (104/290) for the web-based survey and 44% (24/54) for the telephone survey. A total of 110 (n=86, 78.2% web and n=24, 21.8% telephone) surveys were included for quantitative analysis, and 97 (n=74, 76% web and n=23, 24% telephone) surveys were included for direct content analysis. Figures 1 and 2 show a flowchart summarizing the subsequent exclusion of cases from the original number participants to arrive at the final analysis. A total of 97.3% (107/110) of the participants used telephone consultations. The percentages of missing values (1% to 8%) for each Likert-scale question were insignificant (<20%); therefore, the mean of each item was used to replace the missing data [38].

The descriptive statistics of the demographic, clinical, and telemedicine variables are presented in Tables 1-3. Briefly, most of the participants (99/110, 90%) were aged ≥55 years, were retired (80/109, 73.4%) and married (76/110, 69.1%), lived with others (85/109, 78%), and lived within 20 km of where the stroke prevention clinic was located (77/110, 70%). Only a few participants (7/109, 6.4%) had an educational level lower than high school, and most (67/109, 61.5%) had a postsecondary education (Table 1). Regarding clinical factors, most participants had a stroke diagnosis (71/110, 64.5%) and self-identified as having only one stroke risk factor (74/110, 67.3%). Most patients were new (101/110, 91.8%) to the stroke prevention clinics (Table 2). Many participants had relatively less experience with health technology. Although most of them owned digital equipment for teleconsultation (79/109, 72.5%), many of them had never used patient portals (94/108, 87%) and telemedicine (92/108, 85.2%) before the COVID-19 pandemic (Table 3).

**Table 1:** Demographic characteristics of the patients included in the study (N=110).

Variable			Total^a^		Low satisfaction (n=26)		High satisfaction (n=84)		*P* value	
**Age group (y), n (%)**										.04^b^
	<55	11 (10)		2 (7.7)		9 (10.7)				
	55-64	18 (16.4)		3 (11.5)		15 (17.8)				
	65-74	25 (25.5)		4 (15.4)		24 (28.6)				
	75-84	40 (36.4)		16 (61.5)		24 (28.6)				
	≥85	13 (11.8)		1 (3.8)		12 (14.3)				
**Sex, n (%)**										.52
	Female	49 (44.5)		13 (50)		36 (42.9)				
	Male	61 (55.5)		13 (50)		48 (57.1)				
**Distance (km), mean (SD)**			17.73 (16.52)		13.53 (11.65)		19.03 (17.61)		.31	
	1-20, n (%)	77 (70)		21 (80.8)		56 (66.7)				
	21-40, n (%)	21 (19.1)		4 (15.4)		17 (20.2)				
	>40, n (%)	12 (10.9)		1 (3.8)		10 (13.1)				
**Education, n (%)**										.52
	Grade 8 or lower	7 (6.4)		0 (0)		7 (8.3)				
	High school	35 (32.1)		10 (40)		25 (29.8)				
	College	32 (29.4)		6 (24)		26 (31)				
	University	24 (21.8)		6 (24)		18 (21.4)				
	Graduate school	11 (10)		3 (12)		8 (9.5)				
**Employment, n (%)**										.68
	Retired	80 (73.4)		20 (76.9)		60 (72.3)				
	Working	17 (15.6)		2 (7.7)		15 (18.1)				
	Unemployed	4 (3.7)		1 (3.8)		3 (3.6)				
	Self-employed	3 (2.8)		1 (3.8)		2 (2.4)				
	On disability	5 (4.6)		2 (7.7)		3 (3.6)				
**Marital status, n (%)**										.42
	Married	76 (69.1)		18 (69.2)		58 (69.1)				
	Divorced	11 (10)		4 (15.4)		7 (8.3)				
	Widowed	17 (15.5)		4 (15.4)		13 (15.5)				
	Single	6 (5.5)		0 (0)		6 (7.1)				
**Living arrangement, n (%)**										.69
	With others	85 (78)		21 (80.8)		64 (77.1)				
	Alone	24 (22)		5 (19.2)		19 (22.9)				
**Transportation, n (%)**										.24
	Drives	67 (65)		12 (54.5)		55 (67.9)				
	Relies on others	36 (35)		10 (45.5)		26 (32.1)				
Use of a cane or walker (yes), n (%)			30 (27.5)		8 (30.8)		22 (26.2)		.57	
Language barrier (yes), n (%)			10 (9.1)		6 (23.1)		4 (4.8)		.005^c^	
**Hearing impaired (yes), n (%)**			28 (25.5)		9 (34.6)		19 (22.6)		.22	
	Affecting phone conversations (yes)	14 (50)		6 (66.7)		8 (42.1)				
**Vision impaired (yes), n (%)**			33 (30)		8 (30.8)		25 (29.8)		.92	
	Affecting the use of a screen (yes)	16 (50)		5 (62.5)		11 (45.8)		.41		
The survey was consented to by the SDM^d^ (yes), n (%)			53 (49.1)		18 (69.2)		35 (41.7)		.01^b^	
Web survey (yes), n (%)			86 (78.2)		24 (92.3)		62 (73.8)		.046^b^	

^a^Note that the percentages are based on denominators that vary from the overall sample size of 110 because of missing data.

^b^*P*<.05.

^c^*P*<.01.

^d^SDM: substitute decision maker. Indicates that the survey was consented to and answered with the help of an SDM.

**Table 2:** Clinical characteristics of the patients included in the study (N=110).

Variable		Total, n (%)	Low satisfaction (n=26), n (%)	High satisfaction (n=84), n (%)	*P* value	
Had stroke diagnosis		71 (64.5)	17 (65.4)	54 (64.3)	.92	
Had stroke residual deficits		27 (38)^a^	9 (52.9)^b^	18 (33.3)^c^	.15	
New referral		101 (91.8)	25 (96.2)	76 (90.5)	.36	
**Number of stroke risk factors**						.40
	0	14 (12.7)	3 (11.5)	11 (13.1)		
	1	74 (67.3)	21 (80.8)	53 (63.1)		
	2-4	19 (17.3)	1 (3.8)	18 (21.4)		
	5-6	3 (2.7)	1 (3.8)	2 (2.4)		

^a^N=71.

^b^N=17.

^c^N=54.

**Table 3:** Telemedicine-related characteristics of the patients included in the study (N=110).

Variable		Total, n (%)^a^	Low satisfaction (n=26), n (%)	High satisfaction (n=84), n (%)	*P* value	
Used telephone		107 (98.2)	26 (100)	81 (97.6)	.42	
**Number of stroke prevention clinic home-based teleconsultations**						.74
	Once only	52 (47.3)	13 (50)	39 (46.4)		
	2-4 times	50 (45.5)	12 (46.2)	38 (45.2)		
	≥5 times	8 (7.3)	1 (3.8)	7 (8.3)		
**Patient portal use before the COVID-19 pandemic**						.71
	Never	94 (85.5)	23 (92)	71 (85.5)		
	1-2 times	10 (9.1)	2 (8)	8 (9.6)		
	3-5 times	3 (2.7)	0 (0)	3 (3.6)		
	>5 times	1 (0.9)	0 (0)	1 (1.2)		
**Telemedicine use before the COVID-19 pandemic**						.35
	Never	92 (83.6)	21 (80.8)	71 (86.6)		
	1-2 times	7 (6.4)	2 (7.7)	5 (6.1)		
	3-5 times	6 (5.5)	1 (3.8)	5 (6.1)		
	>5 times	3 (2.7)	2 (7.7)	1 (1.2)		
Previsit contact by the stroke prevention clinic (no)		94 (85.5)	24 (92.3)	70 (85.4)	.36	
Owned digital equipment at home (yes)		79 (71.8)	19 (73.1)	60 (72.3)	.94	

^a^Note that the percentages are based on denominators that vary from the overall sample size of 110 owing to missing data.

### Findings From the Quantitative Analysis

Overall, the instrument was reliable as the Cronbach α reliability analysis of the SERVPERF questionnaire was .894 (Multimedia Appendix 7), which indicated an excellent level of reliability of the instrument [39]. The adjusted *R*^2^ value was 0.76, indicating that the 5 SERVPERF dimensions could explain 76% of the variation in the global satisfaction score. The mean global satisfaction score was 2.5 (SD 0.65) for the low-satisfaction group and 4.40 (SD 0.49) for the high-satisfaction group. To examine the explanatory variables that were significantly associated with the binary outcome variable, consent from the substitute decision maker (SDM), language barrier, age group, survey method, and 5 service dimensions were entered into the final forward stepwise logistic regression model. The adjusted *R*^2^ indicated that 69% of the variance could be explained in the final model. Table 4 illustrates that consent from the SDM (adjusted odds ratio [AOR] 6.59, 95% CI 1.45-29.93; *P*=.01) was positively associated (β=1.89) and the responsiveness (AOR 0.03, 95% CI 0.006-0.188; *P*<.001) and empathy (AOR 0.12, 95% CI 0.02-0.80; *P*=.03) dimensions were negatively associated (β=−3.37 for responsiveness; β=−2.15 for empathy) with dissatisfaction with the home-based teleconsultation service quality (Table 4). The odds of dissatisfaction for participants who consented to the survey through their SDM were 6.59 (95% CI 1.45-29.93) compared with those who consented themselves. Every one-unit increase in the responsiveness dimension score decreased the odds of dissatisfaction by 0.03 (95% CI 0.006-0.19) when other variables were held constant. Every one-unit increase in the empathy dimension score decreased the odds of dissatisfaction by 0.12 (95% CI 0.02-0.8) when other variables were held constant.

**Table 4:** The forward stepwise binary logistic regression model.

Variable	β (SE)	*P* value	AOR^a^ (95% CI)
Consent from SDM^b^	1.89 (0.772)	.01^c^	6.59 (1.45-29.93)
Responsiveness^d^	−3.37 (0.867)	<.001^e^	0.03 (0.006-0.19)
Empathy^f^	−2.15 (0.986)	.03^c^	0.12 (0.02-0.80)

^a^AOR: adjusted odds ratio.

^b^SDM: substitute decision maker. Consent from SDM indicates that the survey was answered with the help of an SDM.

^c^*P*<.05.

^d^Responsiveness is a service performance model dimension regarding the willingness to help customers and provide a prompt service.

^e^*P*<.001.

^f^Empathy is a service performance model dimension regarding providing individual care and attention to customers.

The significant characteristics of the participants who had their SDM sign the consent form and help them answer the survey are listed in Table 5. The participants whose SDM provided consent to help them answer the survey were more likely to answer a web-based survey (*χ*^2^_2_=15.6; *P*<.001) and have a language barrier (*χ*^2^_2_=15.6; *P*<.001), hearing impairment (*χ*^2^_2_=3.9; *P*=.048), or hearing that affected telephone conversations (*χ*^2^_2_=5.6; *P*<.02) and were less likely to drive (*χ*^2^_2_=7.0; *P*=.04)*.* Tangibles was the only statistically significant SERVPERF dimension (*P*<.001). The participants who consented through their SDM had a shorter travel distance, were older, and were more likely to have residual stroke symptoms.

**Table 5:** Comparison of variables between surveys for which consent was obtained from the substitute decision maker (SDM) and surveys for which consent was obtained from the patient themselves (difference of >15%).

Characteristic	Consent from SDM^a^ (n=53)	Consent from patient (n=57)	*P* value
Web-based survey, n (%)	50 (94)	36 (63)	<.001^b^
Age (y), mean (SD)	73.75 (13.085)	70.60 (10.61)	.17
Male sex, n (%)	34 (64)	27 (47)	.08
Distance (km), mean (SD)	15.897 (16.525)	19.435 (16.48)	.26
Driving, n (%)	27 (55)^c^	40 (74)^d^	.04^e^
Language barrier, n (%)	10 (19)	0 (0)	<.001^b^
Hearing impaired, n (%)	18 (34)	10 (18)	.048
Hearing impairment affecting phone conversations, n (%)	12 (67)^f^	2 (20)^g^	.02^e^
Residual stroke symptoms, n (%)	17 (49)^h^	10 (28)^i^	.07
Tangibles, mean (SD)	3.21 (0.83)	3.67 (0.61)	<.001^b^
Reliability, mean (SD)	3.87 (0.82)	3.94 (0.56)	.68
Responsiveness, mean (SD)	3.76 (0.94)	3.76 (0.59)	.98
Assurance, mean (SD)	3.99 (0.78)	4.04 (0.53)	.68
Empathy, mean (SD)	3.75 (0.71)	3.96 (0.56)	.34

^a^Consent from SDM indicates that the survey was answered with the help of an SDM.

^b^*P*<.001.

^c^N=49.

^d^N=54.

^e^*P*<.05.

^f^N=18.

^g^N=10.

^h^N=35.

^i^N=36.

### Findings From the Content Analysis

#### Overview

A total of 88.2% (97/110) of patients completed the open-ended questions in the survey, with 25% (24/97) in the low-satisfaction group and 75% (73/97) in the high-satisfaction group. Multimedia Appendices 8 and 9 list the dimensions and subcategories of positive and negative comments among patients with low and high satisfaction scores, respectively. Interestingly, the low- and high-satisfaction groups shared the same dissatisfied service dimensions (assurance, reliability, and empathy) and subcategories. Overall, missing clinical components, inadequate communication, administrative issues, and absence of personal connection were the significant concerns that affected patients’ perceived home-based teleconsultation quality at the stroke prevention clinics.

In contrast, the most satisfying service dimensions were assurance, empathy, and responsiveness among the high-satisfaction group. Overall, a competent clinician with effective communication skills and great empathy for patients is crucial for patient-perceived high-quality care in home-based teleconsultation. In addition, convenience, appropriateness to the patient’s situation, and timely consultation were important for high satisfaction with home-based teleconsultation.

#### Future Use of Home-Based Teleconsultation

Assessment of patient preference for future use of home-based teleconsultation under normal circumstances (after the COVID-19 pandemic) showed that 35% (33/95) of participants preferred not to use home-based teleconsultation. Most participants (18/23, 78%) in the low-satisfaction group preferred not to use home-based teleconsultation. In contrast, 56% (40/72) of participants in the high-satisfaction group were willing to use it, and 24% (17/72) indicated that they might use home-based teleconsultation for specific reasons. However, a minority of participants in the high-satisfaction group (15/72, 21%) still refused to use it in normal circumstances, with primary concerns of communication issues, lack of personal connection, and the belief in the superiority of in-person consultations.

## Discussion

### Principal Findings

Home-based teleconsultation as a form of telemedicine rapidly expanded in many health sectors in Canada during the COVID-19 pandemic owing to lockdowns and social distancing restrictions [31]. Since the COVID-19 pandemic, home-based teleconsultation has become essential in outpatient service delivery [40]. By April 2020, 77% of Ontario ambulatory visits were conducted using teleconsultation, a total of 77% of ambulatory visits were conducted using a virtual modality [41]. Nearly all (32/33, 97%) Ontario stroke prevention clinics that responded to a province-wide survey from June 2021 to July 2021 (response rate of 33/41, 80%) reported that they had adopted home-based teleconsultation as a service delivery mode in addition to in-person visits since the COVID-19 pandemic [42]. Patient satisfaction should be the priority in future virtual care development and adoption [43]. To our knowledge, this is the first study to use a service quality theoretical lens to assess patient satisfaction with home-based teleconsultation in outpatient stroke care during the pandemic. Many studies of patient surveys during the COVID-19 pandemic have found that most patients and clinicians reported positive experiences with teleconsultation at outpatient neurology services during the COVID-19 pandemic [31]. However, no study has investigated outpatient stroke prevention services or examined the service quality of home-based teleconsultation during the COVID-19 pandemic. The patient population of stroke prevention clinics and the disease characteristics differ from those of other chronic neurological diseases. For instance, patients at stroke prevention clinics have a unique mix of acuity (such as early identification of large vessel occlusion and cardiac source of embolism) and chronic disease management (eg, hypertension, dyslipidemia, diabetes, and lifestyle management), and most are older adults. Owing to health resource disparity, timely access to outpatient magnetic resonance imaging is not always feasible for minor strokes. When referred to stroke prevention clinics, patients with TIA have transient neurological symptoms, unremarkable brain images, and normal physical examinations. History taking is essential in patients with TIA. The unique patient population and characteristics may pose different challenges in home-based teleconsultation, especially for newly referred patients. Our study found that the participants who were older (mean age 72.12, SD 11.92 years) and mostly newly referred (101/110, 91.8%) and used the telephone modality (107/109, 98.2%) were satisfied with the home-based teleconsultation provided by the stroke prevention clinics during the COVID-19 pandemic. We identified patient-reported factors that affected their satisfaction with the service quality of home-based teleconsultation. Our study filled these research gaps.

Responsiveness was the most statically significant dimension in our quantitative results and is an influential factor for a positive experience. Convenience was the main subtheme of the responsiveness dimension in the high-satisfaction group. The patients in the low-satisfaction group tended to live closer to the stroke prevention clinics, with an average distance of 13.53 (SD 11.57) km, than those in the high-satisfaction group (mean 19.03, SD 17.61 km). Even though they were less likely to drive and had some communication barriers (more likely to have language barriers or hearing impairments), convenience was not a positive factor influencing their satisfaction. Our content analysis supported that convenience, by saving time, travel, and energy, influenced patients’ positive perceptions of the personal benefits of home-based teleconsultation during the pandemic [31]. Our findings were consistent with those of studies conducted before the COVID-19 pandemic [44]. A systematic review of digital experience also found that convenience is one of the motivating factors contributing to a positive digital patient experience [45]. The convenience of home-based teleconsultation is an influential factor in swaying patients’ satisfaction with service quality to the positive side and their preference for teleconsultation [31]. However, convenience is not equivalent to good quality of care. We need to consider the effect of convenience when assessing patients’ preferences and evaluating patient satisfaction with the service quality of home-based teleconsultation.

Second, the empathy dimension was a significant factor in both the quantitative and qualitative analyses. Dissatisfaction feedback in the empathy dimension was found in both the low- and high-satisfaction groups. Our study participants used mostly the telephone modality, where the lack of nonverbal cues may be associated with a profound concern about the lack of personal connection, which is the primary subcategory of the empathy dimension. As indicated by existing studies, the replacement of interpersonal connection and a lack of physical human contact are negatively associated with digital patient experiences [45]. The literature shows that empathy can have powerful effects on positive patient outcomes and satisfaction [46,47]. There is a concern that the digitalization of health care services could primarily lead to a decrease in the expression of empathy [46]. A study on patient satisfaction with tele-obstetric care found that a desire for personal connection via face-to-face interaction with a clinician was a critical motivation for selecting in-person versus teleconsultation care modalities [48]. Our findings are in line with those of the previous literature. The lack of personal connection in our content analysis could explain the negative relationship between the empathy dimension and dissatisfaction. An interesting finding from our content analysis was that even 98% (81/83) of the participants in the high-satisfaction group used the telephone modality; they expressed overwhelmingly more positive than negative comments (34 vs 12) on the empathy dimension. The clinician’s empathy skills may significantly enhance patient experiences even with a low-technology modality. Empathy skill training for clinicians, primarily through computer-mediated communications, is a key area to study in the future [46].

Third, although the assurance dimension was not found to be statistically significant between the low- and high-satisfaction groups, this was likely due to no real difference between the 2 groups. Our content analysis indicated that the assurance dimension was one of the most important SERVPERF dimensions in both the low- and high-satisfaction groups. The subcategories raised from the content analysis revealed that the patients’ concerns in the assurance dimension were the missing clinical components—especially physical examination—and inadequate communication. The view of the inferior quality of a remote examination among clinicians was dominant in outpatient neurology teleconsultation before the COVID-19 pandemic [49]. This is likely why most teleconsultations were performed for follow-up patients with chronic neurological diseases before the COVID-19 pandemic [50]. Compared with before the COVID-19 pandemic, home-based teleconsultation has been widely used in both new and follow-up patients at home without the luxury of having a health care professional assisting a teleconsultation since the COVID-19 pandemic [31]. The lack of a physical examination and inadequate communication could impede the clinician’s ability to diagnose and formulate a treatment plan, especially for new patients [31]. In addition, the use of video is more challenging than the use of a telephone because of the rapid adaptation and lack of preparation. According to the Ontario stroke prevention clinic web survey, nearly half of the clinics use only the telephone [42]. Video consultations enable a certain degree of remote examination and may facilitate better communication, whereas telephone-only visits limit clinical assessment and communication. Telephone consultations lack body language and physical prompts, which could negatively affect the communication between the clinician and patient. However, most of the participants in our study used telephone consultations (107/110, 97.3%), and their overall satisfaction was high (3.9/5). Future studies could consider patient satisfaction when using a telephone-only modality for new referrals in this patient population under normal circumstances.

The appropriateness of patient selection is a critical factor in high-quality home-based teleconsultation from patients’ perspectives. Our statistical findings indicated that patients who required help from their SDM to consent and answer the survey were positively associated with dissatisfaction. The SDM may have chosen a web survey, as the participants had difficulty answering a telephone survey because of language barriers, hearing impairments, or communication difficulties from residual stroke symptoms (such as aphasia, apraxia, or mild cognitive impairment; Table 5). Moreover, the patients who needed their SDM to consent and help them answer the survey scored significantly lower in the tangibles dimension, which may indicate that the participants had low comfort levels with technology and technical difficulties even with the telephone modality. The consent from an SDM has many unknown characteristics and requires further exploration in future research.

Similarly, the analysis showed that there were no statistically significant differences in the reliability dimension between the low- and high-satisfaction groups. However, it is important to note that issues primarily related to administration, which fall under the reliability dimension, had a negative impact on both the low- and high-satisfaction groups. This possibility aligns with findings from a scoping review that the lack of proper administrative support has harmed clinicians’ teleconsultation satisfaction [31]. Our findings indicate that it also negatively affects patient satisfaction. Some of the low satisfaction may be due to the abrupt change to teleconsultation because of the COVID-19 pandemic and the lack of clinical and patient preparation. We know that some administrative problems are not unique to home-based teleconsultation, as they occur during in-person visits. Home-based teleconsultation may have increased the workload by keeping pace with the transitioning workflow among telephone, video, and in-person visits, contributing to the maladaptation of home-based teleconsultation [51]. Establishing a new care pathway for home-based teleconsultation may streamline the administrative workflow.

The assurance, empathy, and reliability dimensions all had the most negative comments in both the low- and high-satisfaction groups, and the high-satisfaction group had the most positive remarks in the assurance and empathy dimensions in the content analysis, which showed a double-edged effect. This finding might indicate that different key subcategories have buffer effects on improving patient satisfaction with the service quality of home-based teleconsultation. This may reflect the advantage of using both quantitative and qualitative data to provide diverse types of information [37]. By comparing them side by side, the qualitative analysis may provide insights to explain the quantitative findings.

### The Future of Stroke Prevention Clinics’ Service Delivery Mode

A combination of teleconsultation and in-person visits for outpatient stroke prevention care is the future. Our study showed that 45% (43/95) of participants were willing to use and 18% (17/95) would consider using home-based teleconsultation in future nonpandemic conditions. A study examining patient preference for telehealth for nonemergent health issues after the COVID-19 pandemic concluded that patients were generally willing to use video but preferred in-person visits [52]. A patient-centered service should be delivered by offering the patient a choice [20]. Virtual care provides an opportunity to design a health system that is actually patient-centered [43]. A combination of in-person visits and home-based teleconsultation—a hybrid care model—could best meet patient needs by improving efficiency and capacity without added risk [53,54].

Hybrid care should be sustainable in practice settings to ensure patient care quality, equity, and justice [53]. To avoid increasing the digital divide, a telephone may be favorable instead of video calls for older patients and those with a lower education or income and from racial and ethnic minority groups [55]. However, we should refrain from creating a 2-tiered health care system in which high-income individuals receive video consultations and low-income individuals receive phone consultations. Patients should receive the right care in the right setting, at the right time, and with the right mode; the cost of the service should be reduced; and the best clinical practice guidelines should be followed [56]. Hybrid care could be a balanced approach to achieving a high-performance health care system. Patients can choose the best model by considering flexible options, and clinicians can offer individualized recommendations for optimal care modalities [54].

### Limitations

Our study has several limitations. First, the cross-sectional survey only provides a snapshot of a phenomenon and cannot determine the temporal relationship between the dependent and independent variables. Second, the participants in this study may not be generalizable to patients of other stroke prevention clinics in Ontario, notably in areas with different health resources such as urban versus very remote rural centers. In addition, the open-ended responses to the web survey were very brief, limiting our ability to gain a deeper understanding of their experiences. Next, as we surveyed patients who had had a home-based teleconsultation within 6 months, recall bias is possible [57]. Only patients who had a home-based teleconsultation in January 2021 and February 2021 received the survey 4 to 5 months later; the following patients received their survey 2 and a half months after the home-based teleconsultation on average (range 1-3 months). In addition, there is potential nonresponse bias, as web surveys usually have a low response rate [57]. A meta-analysis comparing web survey response rates concluded that the average response rate for web surveys was approximately 11% [58]. Our web survey had a 35.9% (104/290) response rate, and the telephone survey yielded a 44% (24/54) response rate.

Overall, although our study may only reflect part of the concept of satisfaction with service quality because of its complexity, it provided substantial insights into areas for quality improvements from patients’ point of view. The literature suggests that patient satisfaction is a multidimensional concept that still needs to be fully defined. The patient satisfaction scores may reflect the demographic mix and clinical and psychological picture of the patients served by a medical service [59]. Our study attempted to use a theory-guided quantitative and qualitative analysis to reveal the relationship and explanation of such a complex phenomenon. Despite the problems of using patient satisfaction to assess service quality, its measurement provides unique information regarding the care process as seen through the patients’ eyes [59]. Patients still provide the best source of accurate information on the care they receive [60]. In the absence of choice in public-funded health care, our survey gave patients a voice to indicate their preferences [21].

### Conclusions

Our findings highlighted 2 crucial service quality dimensions (responsiveness and empathy) that were negatively statistically significantly associated with patient dissatisfaction. Moreover, we identified that a survey consented to by an SDM was positively associated with dissatisfaction. In addition, there were 4 subcategories related to patient dissatisfaction (missing clinical activities, inadequate communication, administrative process issues, and absence of personal connection). We anticipate that appropriate patient selection, consideration of patient preferences, a streamlined home-based teleconsultation administrative workflow, and a competent clinician with communication and empathy skills are essential for achieving high satisfaction with home-based teleconsultation. These factors could be considered when designing home-based teleconsultation services to enhance patient experiences of stroke prevention care.

## Appendix

Multimedia Appendix 1 Survey consent sheet for participants.

Multimedia Appendix 2 Cover letter for mail-in package.

Multimedia Appendix 3 The description of 18 items used in the service performance model questionnaire.

Multimedia Appendix 4 Survey: the patient’s perception of teleconsultation service quality at stroke prevention clinic during COVID-19.

Multimedia Appendix 5 Six questions to check the clarity of the survey questions.

Multimedia Appendix 6 Definitions of 10 demographic, 7 clinical, and 6 technical-related independent variables.

Multimedia Appendix 7 Cronbach α reliability statistics and item-total statistics of the service performance model questionnaire.

Multimedia Appendix 8 Positive and negative categories among patients with low global satisfaction.

Multimedia Appendix 9 Positive and negative categories among patients with high global satisfaction.

## Abbreviations

AOR: adjusted odds ratio
REDCap: Research Electronic Data Capture
SDM: substitute decision maker
SERVPERF: service performance model
SERVQUAL: service quality model
TIA: transient ischemic attack

## Data Availability

Data are available upon request from the authors.
