# Supplementary material for: Factors That Influence Patient Satisfaction With the Service Quality of Home-Based Teleconsultation During the COVID-19 Pandemic: Cross-Sectional Survey Study
Source: JMIR Cardio. 2024 Feb 16;8:e51439. doi: 10.2196/51439 (PMC10907934; doi:10.2196/51439)
Supplement: Multimedia Appendix 1 [file cardio_v8i1e51439_app1.docx]

**Multimedia Appendix 1**

Survey Consent Sheet for Participant

Researchers from the University of Waterloo are conducting a study to learn your perception on your virtual consultation with the Stroke Clinic at Southlake Regional Health Center since the start of the COVID-19 pandemic. We would like to understand your experiences with the service quality at the Stroke clinic during this unprecedented time.

We are interested in your views and your experiences during the virtual consultation, both positive and negative.

The survey includes a few demographic questions, a questionnaire, and open-ended questions. It should take approximately 15 minutes to complete. Participation in the project is voluntary. You may skip any questions you do not wish to answer, and you may withdraw from the study at any time by expressing this to the researcher during the survey.

Your identity will be considered confidential. Your name will not be included in any presentations or publications arising from this project. Your interview will be audio recorded to ensure an accurate recording of your responses. The data collected from the telephone survey will be kept on a secure server hosted in the Southlake Regional Health Center.

There is the potential for risks or discomfort associated with participation since the questions may ask you to recall a time that was stressful or distressing. You may exit the telephone survey at any time by expressing this to the researcher and your data will not be submitted. Once submitted, data cannot be withdrawn.

We cannot promise any personal benefits to you from your participation in this study. However, the information you share with us will be used to understand the impact of the service interruptions in outpatient clinic services quality by COVID-19 from the patients’ perspective. This information will help us to identify strategies on how to maintain the service quality in a future pandemic situation. We appreciate your participation and contributions.

Some demographic questions such as income, education, marital status, and ethnicity helps to describe the participants’ characteristics in the study.

An example of the type of open-ended questions asked in the survey is:

What are the main challenges in using telemedicine services during COVID 19 lockdown?

Your name will not be collected in the survey, and your data will be grouped with other participants’ data, separate from any identifying information. The data will be stored for a minimum of 7 years.

This study has been reviewed and has received exemption from Southlake Research Ethics Board. Southlake Hospital will receive summary data from the study to use for quality improvement purposes. This study has also been reviewed and cleared by the University of Waterloo Research Ethics Committee (ORE #42686). If you have questions for the committee, contact the Office of Research Ethics, at 1-519-888-4567 ext. 36005 or [ore-ceo@uwaterloo.ca](mailto:ore-ceo@uwaterloo.ca).

Thank you in advance for your interest in this project. I agree to participate in the research study as described. I understand the purpose and nature of this study and I am participating voluntarily. I understand that I can withdraw from the study at any time, without any penalty or consequences.

Check on the “Yes” below indicates that I have read the above information and voluntarily agree to participate. By agreeing to participate in the study, I am not waiving my legal rights or releasing the investigator(s) or involved institution(s) from their legal and professional responsibilities.

1. Yes___
2. No___ 
    
   If the substitute decision-maker is consenting for the participant: 
    
   By checking “Yes,” you consent that you are willing to help the participant to answer the questions in this survey.
3. Yes__
4. No__

If you have questions, please contact Guangxia Meng by email [g3meng@uwateloo.ca](mailto:g3meng@uwateloo.ca) or by phone at 905-895-4521 extension XXXX.
