# Supplementary material for: Factors That Influence Patient Satisfaction With the Service Quality of Home-Based Teleconsultation During the COVID-19 Pandemic: Cross-Sectional Survey Study
Source: JMIR Cardio. 2024 Feb 16;8:e51439. doi: 10.2196/51439 (PMC10907934; doi:10.2196/51439)
Supplement: Multimedia Appendix 2 [file cardio_v8i1e51439_app2.docx]

**Multimedia Appendix 2**

Cover letter for mail-in package

Hello,

My name is Guangxi Meng, and I am a PhD's student working under the supervision of Dr. Helen Chen and Dr. Carrie McAiney in the school of Public Health and Health System at the University of Waterloo. As part of my PhD's degree, I am conducting a research study on patient's perceptions of teleconsultation service quality at a stroke prevention clinic. Given your experiences, I feel that you are well suited to provide insight into this topic, and I would like to invite you to participate in this study.

I am sending this letter as you expressed the interest in participating in the survey. Your participation will consist of a one-on-one telephone survey and interview that will take approximately 15-20 minutes of your time. During the interview you will be asked questions such as "what are the main challenges in using teleconsultation services during COVID 19 lockdown?" With your permission, I would like to audio-record the interview to ensure accurate transcription and analysis.

I would like to assure you that the study has been reviewed and received ethics clearance through a University of Waterloo Research Ethics Board (#42686). It is also a quality improvement project at Mackenzie Health and received ethics exemption. However, the final decision about participation is yours.

This package includes a telephone survey consent and the questionnaires. This package is not intended for you to fill out; instead, it offers you the opportunity to read and understand the consent and the study thoroughly before arranging a telephone call. I will call you within a week to see if you are interested in the telephone survey. A researcher from the University of Waterloo will contact you and will complete the survey by phone. She will also ask a few open-ended questions. If you require ire additional information to assist you in reaching a decision about participation, please do not hesitate to contact me at 905-895-4521 ext. XXXX or by email g3meng@uwaterloo.ca, also contact my supervisor at 519-888-4567 ext. 42132 or by email hele.chen@uwaterloo.ca.

You sincerely

Guangxia
