# Supplementary material for: Factors That Influence Patient Satisfaction With the Service Quality of Home-Based Teleconsultation During the COVID-19 Pandemic: Cross-Sectional Survey Study
Source: JMIR Cardio. 2024 Feb 16;8:e51439. doi: 10.2196/51439 (PMC10907934; doi:10.2196/51439)
Supplement: Multimedia Appendix 3 [file cardio_v8i1e51439_app3.docx]

**Multimedia Appendix 3**

The description of 18-items used in the Service Performance Model questionnaire

| Dimension description | Item description |
| --- | --- |
| **Tangible:**  Technical and the home environment experiences | 1. Comfort level using virtual equipment  2. Technical difficulties during the teleconsult  3. The effect of home environment |
| **Reliability:**  ability to perform the promised service responsibly and accurately | 4. The clinic informs and prepares the patient before the visit  5. The clinic offers the service inadequate time  6. The clinic provides its services at the time it promises to do so |
| **Responsiveness**:  willingness to provide help and a prompt service to customers | 7. The teleconsult is convenience for the patient.  8. The clinic is easy to be contacted by the patient  9. The clinic prompt responds to the patient  10. The clinic has provided the services as they promised to meet the medical needs |
| **Assurance**:  the knowledge and courtesy of the clinicians and their ability to inspire trust and confidence | 11. The patient understood the advice  12. The clinician has the knowledge-making diagnosis  13. The behaviour of the clinician instills confidence in the patient  14. The patient feel able to understand the conversation easily  15. The behaviour of the clinician instills trust in the patient |
| **Empathy**  Caring and understanding which provides and/or offers its customers in terms of its individualized and personalized attention | 16. The staff provide service in a friendly and courteous way  17. The clinician listens to the patient  18. The patient feels personal attention |
