# Supplementary material for: Factors That Influence Patient Satisfaction With the Service Quality of Home-Based Teleconsultation During the COVID-19 Pandemic: Cross-Sectional Survey Study
Source: JMIR Cardio. 2024 Feb 16;8:e51439. doi: 10.2196/51439 (PMC10907934; doi:10.2196/51439)
Supplement: Multimedia Appendix 4 [file cardio_v8i1e51439_app4.docx]

**Multimedia Appendix 4**

Survey: The patients’ satisfaction of teleconsultation service quality at stroke prevention clinics during the COVID-19 pandemic

1. The first set of questions are about you.
2. How old are you? _____
3. What gender do you identify with

- Man/Male
- Woman/Female
- Trans* / Non-binary
- Prefer to self-identify (option to specify)
- Prefer to not answer

1. Were you diagnosed with a stroke or mini-stroke (TIA) by the clinic?

- Yes __

If yes, do you have any disability (such as weakness, numbness, speech, vision problems) left from this event? Yes____, No____

- No __

1. At the time of your first teleconsultation (consultation by phone or video) at the Stroke prevention clinic, were you a new patient at the clinic?

- Yes __
- No __

1. How many teleconsultations (by phone or video) did you have in total at the stroke prevention clinic? _____
2. Please check all medical conditions that you currently have been diagnosed or taking medication for:

- High blood pressure ___
- High cholesterol  ___
- Diabetes (high blood sugar) ____
- Heart disease____
- Previous stroke ____
- Smoker____
- Vascular disease (blood vessel plaques or narrowing)____
- Others____

1. What is the highest level of education you have completed :

- Grade 8 and below__
- High School__
- College__
- University__
- Graduate Degree__

1. Employment during COVID-19

- Retired__
- Working__
- Unemployed__
- Self-employed__
- On disability__

1. Marital status

- Married__
- Single__
- Widowed__
- Divorced/separated__

1. Living situation

- Alone__
- Living with others

1. How far do you live from the hospital? Please enter a distance in kilometers (KM)____
2. Type of transportation most you commonly used for clinic visits prior to the COVID-19 pandemic:

- Self
- rely on others or public transportation

1. Do you need any assistance with walking (use of a cane, walker, wheelchair, or the support of another person)?

- No
- Yes

1. Do you have any difficulty understanding and speaking English during a medical visit?

- Yes
- No

      H. Do you have hearing loss?

- No
- Yes, ___ Does it affect your ability to hear people when you are talking to them on the telephone? YES,__ NO__

1. Do you have vision loss?

- No
- Yes,____ Does it affect your ability to use electronic devices such as an iPad or computer Yes___ NO__

1. Please think back to your teleconsultation experiences:

1. Was your most recent teleconsultation at the stroke prevention clinic done by telephone or video?

- telephone
- video

1. Before COVID-19, did you ever use the patient connect portal? If so, how often did you use it before COVID-19?

- None
- A little (used 1-2 times)
- Some (used 3-5 times)
- A lot (used more than 5 times)

1. Before COVID-19, did you ever have a teleconsultation? If so, how often did you use it before COVID-19?

- None
- A little (used 1-2 times)
- Some (used 3-5 times)
- A lot (more than 5 times)

1. Before your teleconsultation, did the clinic contact you on how to use the video appointment function?

- YES____
- NO_____

1. Do you have the equipment for virtual visits at home (such as a tablet or mobile phone with Wi-Fi, camera, and speaker)?

- YES___
- NO____

1. The Modified SERVPERF questionnaire

**How would you evaluate the most recent teleconsultation you have received from the stroke prevention clinic during the COVID-19 pandemic?**

**Using the scale below, please rate your experiences with each of the following:**

**SD = strongly disagree**

**D = disagree**

**N = neither disagree nor agree**

**A = agree**

**SA = strongly agree**

|  | **Question** | **SD** | **D** | **N** | **A** | **SA** |
| --- | --- | --- | --- | --- | --- | --- |
| 1 | I am comfortable using the virtual equipment (phone, iPad or computer) on my own. |  |  |  |  |  |
| 2 | I have experienced technical difficulties during the visit. |  |  |  |  |  |
| 3 | Being at home for my visit makes me feel more relaxed. |  |  |  |  |  |
| 4 | The stroke prevention clinic staff contacted me to prepare for the visit. |  |  |  |  |  |
| 5 | My virtual visit started on time. |  |  |  |  |  |
| 6 | The length of the visit is about right. |  |  |  |  |  |
| 7 | Having a teleconsultation is convenient for me |  |  |  |  |  |
| 8 | The stroke prevention clinic staff were easy to contact. |  |  |  |  |  |
| 9 | The stroke prevention clinic staff quickly responded to my questions and concerns. |  |  |  |  |  |
| 10 | I feel all my needs and questions were addressed during the visit. |  |  |  |  |  |
| 11 | I understood the advice that I received during the visit. |  |  |  |  |  |
| 12 | The doctor or nurse practitioner was competent in making diagnoses during my visit. |  |  |  |  |  |
| 13 | I trusted the doctor or nurse practitioner during the visit. |  |  |  |  |  |
| 14 | During the visit, I was able to follow the conversation easily. |  |  |  |  |  |
| 15 | I am confident in the care I received during this visit. |  |  |  |  |  |
| 16 | The stroke prevention clinic staff provide the service in a friendly and courteous way. |  |  |  |  |  |
| 17 | I felt listened to during the visit. |  |  |  |  |  |
| 18 | The lack of personal contact affected my experiences with the visit. |  |  |  |  |  |
| 19 | Overall, I was satisfied with the quality of the service at the stroke clinic |  |  |  |  |  |

1. Semi-structured interview questions:

1. What three words or phrases come to mind when you think about your experience with teleconsultation at the secondary stroke prevention clinic?

1. What difficulties did you have in using the teleconsultation at the stroke prevention clinic?

1. Did you feel the care you received through teleconsultation was as good as seeing the healthcare provider in person at the stroke prevention clinic? Please explain.

1. What clinical activities do you wish to be carried out in person?

1. When COVID 19 pandemic passes and thing goes back to normal, will you be interested to continue using the teleconsultation service?

1. Is there anything the stroke prevention clinic staff can do to improve your experience with teleconsultation – or whatever term you use?

The COVID-19 pandemic has been very difficult for many people. If you need to speak with someone about your feelings, please reach out to your family doctor. You also can reach out to Southlake Regional Health Center Office of Patient Experience at 1-905-895-4521 ext. 2290. If you were contacted by Mackenzie Health, the Patient Relations office is at 1-905-883-1212 ext. 7494.

Thank you for participating in our patient perceptions of stroke prevention clinic service quality during COVID-19 survey! Your feedback is extremely valuable.

If you indicated on the survey that you would like a summary of the results, you can contact me by phone or email, they will be sent to you at the end of 2021.

This study has been reviewed and received ethics clearance through the University of Waterloo Research Ethics Committee (ORE#42686). If you have questions for the committee, contact the Office of Research Ethics at 1-519-888-4567 ext. 36005 or ore-ceo@uwaterloo.ca.

For all other questions or general comments or questions related to this study, please contact Guangxia Meng by email at g3meng@uwateloo.ca or by phone at 905-895-4521, ext. XXXX.
