# Supplementary material for: Factors That Influence Patient Satisfaction With the Service Quality of Home-Based Teleconsultation During the COVID-19 Pandemic: Cross-Sectional Survey Study
Source: JMIR Cardio. 2024 Feb 16;8:e51439. doi: 10.2196/51439 (PMC10907934; doi:10.2196/51439)
Supplement: Multimedia Appendix 5 [file cardio_v8i1e51439_app5.docx]

**Multimedia Appendix 5**

**Six questions to check the clarity of the survey questions**

From your point of view:

- 1. Are the questions clearly articulated? Yes/No. If not, which questions are problematic?
  2. Are the response options relevant to you? Yes/ No. If not, which questions are not appropriate?
  3. Are the response options comprehensive? Yes/Not. If not, Which question needs to be more thorough?
  4. Does the language contain any obscure terminology or ambiguous words? Yes/No, If yes, which questions?
  5. Do you need help understanding the questions? Yes/No. If yes, which question?
  6. How long does it take to finish the survey?
