# Supplementary material for: Factors That Influence Patient Satisfaction With the Service Quality of Home-Based Teleconsultation During the COVID-19 Pandemic: Cross-Sectional Survey Study
Source: JMIR Cardio. 2024 Feb 16;8:e51439. doi: 10.2196/51439 (PMC10907934; doi:10.2196/51439)
Supplement: Multimedia Appendix 6 [file cardio_v8i1e51439_app6.docx]

**Multimedia Appendix 6**

Ten demographic, seven clinical and six technical-related independent variables definitions

| **Independent variables** | **Description** |
| --- | --- |
| **Demographic**  Age  Gender  Distance  Education    Marital status  Living arrangement  Transportation  Mobility  Language barrier  Hearing impairment  Visual impairment  Survey consent by substitute decision-maker  Wen-survey | Years  Female and male  Kilometers from patients’ residence to the stroke prevention clinic  Self-reported less than high school, high school, college, university or graduate study  Self-reported married, divorced, widowed, or single  Self-reported living with others or alone  Self-reported still driving or relying on others  Self-reported independent or using a cane or walker  Self-reported having any difficulty understanding and speaking English  Self-identified having a hearing loss  Self-identified having a vision loss  indicated that the survey was consented to and answered with the help of a substitute decision-maker  The survey was done via web-based method |
| **Clinical**  Stroke diagnosis  Residual symptoms    Stroke risk factors | Self-reported was diagnosed with a stroke by the stroke prevention clinic  Disability such as weakness, numbness, speech, vision problems) left from this event  Medical diagnosis of high blood pressure, high cholesterol, diabetes, heart disease, smoking, vascular disease (blood vessel plaques or narrowing), previous stroke. |
| **Telemedicine-related**  Number of stroke prevention clinic Teleconsult  Portal use before COVID    Telemedicine use before COVID  Pre-visit contact by the stroke prevention clinic  Owning virtual equipment at home | Self-reported numbers of consultations via telephone or video were done with the stroke prevention clinic  Self-reported the usage of a patient-connect portal within the electronic medical record platform offered by the hospital of the stroke prevention clinic before COVID  Self-reported usage of teleconsultation before COVID    Self-reported if the clinic contacted on how to use the video appointment function before the teleconsultation.  Self-reported owning equipment for virtual visits at home such as a tablet or mobile phone with Wi-Fi, camera, and speaker |
