# Supplementary material for: Factors That Influence Patient Satisfaction With the Service Quality of Home-Based Teleconsultation During the COVID-19 Pandemic: Cross-Sectional Survey Study
Source: JMIR Cardio. 2024 Feb 16;8:e51439. doi: 10.2196/51439 (PMC10907934; doi:10.2196/51439)
Supplement: Multimedia Appendix 7 [file cardio_v8i1e51439_app7.docx]

**Multimedia Appendix 7**

Cronbach α reliability statistics and Item-total Statistics of the SERVPERF questionnaire

| Cronbach’s Alpha | | | # of items | | |
| --- | --- | --- | --- | --- | --- |
| 0.89 | | | 18 | | |
| Item number | Scale means if item deleted | Scale variance if item deleted | | Corrected item-total correlation | Cronach’s Alpha if item deleted |
| Question 1 | 65.74 | 99.84 | | 0.17 | 0.91 |
| Question 2 | 65.47 | 102.19 | | 0.16 | 0.9 |
| Question 3 | 65.72 | 96.9 | | 0.41 | 0.89 |
| Question 4 | 65.40 | 96.68 | | 0.45 | 0.89 |
| Question 5 | 65.08 | 98.71 | | 0.54 | 0.89 |
| Question 6 | 65.10 | 96.59 | | 0.62 | 0.89 |
| Question 7 | 65.29 | 92.55 | | 0.66 | 0.88 |
| Question 8 | 65.58 | 93.55 | | 0.59 | 9.89 |
| Question 9 | 65.31 | 94.11 | | 0.65 | 0.88 |
| Question 10 | 65.23 | 91.37 | | 0.85 | 0.88 |
| Question 11 | 65.05 | 95.42 | | 0.72 | 0.88 |
| Question 12 | 65.14 | 94.45 | | 0.72 | o.88 |
| Question 13 | 64.99 | 96.73 | | 0.7 | 0.88 |
| Question 14 | 65.12 | 96.32 | | 0.58 | 0.89 |
| Question 15 | 65.10 | 93.46 | | 0.78 | 0.88 |
| Question 16 | 64.75 | 97.55 | | 0.65 | 0.89 |
| Question 17 | 64.95 | 95.12 | | 0.71 | 0.88 |
| Question 18 | 66.05 | 98.1 | | 0.34 | 0.9 |
