# Supplementary material for: Factors That Influence Patient Satisfaction With the Service Quality of Home-Based Teleconsultation During the COVID-19 Pandemic: Cross-Sectional Survey Study
Source: JMIR Cardio. 2024 Feb 16;8:e51439. doi: 10.2196/51439 (PMC10907934; doi:10.2196/51439)
Supplement: Multimedia Appendix 8 [file cardio_v8i1e51439_app8.docx]

**Multimedia Appendix 8**

Positive and negative categories among patients with low global satisfaction (score of ≤3; N=24).

| Dimension | Positive feedback | | Negative feedback | |
| --- | --- | --- | --- | --- |
|  | Number of participants | Subcategory and example quotes | Number of participants | Subcategory and example quotes |
| Assurance:  the knowledge and courtesy of employees and their ability to inspire trust and confidence | 3 | - Informative - “Good review of my medical situation, informative.” [R57] | 26 | - Missing clinical components (n=15)   - “I feel there is something missing over the phone with a medical diagnosis.” [R21]   - “The doctor can control and check you better.” [R73]   - “Decision Decisions made were good but needed more information to make complete decisions.” [R90] - Inadequate communication (n=11)   - “Can’t discuss needs properly.” [R11]   - “Too hard to explain myself.” [R12]   - “Things I could not explain but could show was not possible.” [R21]   - “It is hard to describe your problem, you can talk with her by your body and face too.” [R73] - Lack of rapport with the clinician (n=2) - “Very artificial, because you can talk, talk, talk on the phone, but lose credibility.” [R77] |
| Reliability: ability to perform the promised service responsibly and accurately | 0 | NA | 13 | - Lack of follow-up booking (n=6)   - “Didn’t have trouble with the appointment itself, but didn’t seem to follow through on anything they were supposed to be doing other than the MRI^a^. Didn’t get test results.” R32] - Wait time (n=4)   - “Most of the time the waiting period isn’t guaranteed, but they try to be on time. Plus the short-staffing, it’s very very hard.” [R77] - Administrative process issues (n=3)   - “Missed a call by two minutes and did not get an appointment again.” [R27] |
| Empathy:  caring and understanding, which a company provides or offers its customers in terms of its individualized and personalized attention | 1 | - Polite - “Polite.” [R85] | 10 | - Lack of personal connection (n=10)   - “Didn’t feel any personal connection.” [R12]   - “Acted like they weren’t interested in me.” [R32]   - “Remote, impersonal.” [R97] |
| Tangibles: technical and home environment experiences | 0 | NA | 4 | - Concerned with phone visit (n=3) - “Miss the in person or video view when talking. Only was by phone.” [R45] - Connectivity issue (n=1) - “Did not connect.” [R17] |
| Responsiveness: willingness to provide help and a prompt service to customers | 2 | - Convenient and prompt - “Convenient for me.”[R79] - “Prompt at first.” [R27] | 3 | - Not responding to calls (n=3) - “[Need to] call people back when they leave phone messages.” [R32] |
| Overall impression | 0 | NA | 10 | - Negative - “Confusing tiring trying.” [R10] - “Terrible, frustrating.” [R12] |

^a^MRI: Magnetic resonance imaging.
