# Supplementary material for: Factors That Influence Patient Satisfaction With the Service Quality of Home-Based Teleconsultation During the COVID-19 Pandemic: Cross-Sectional Survey Study
Source: JMIR Cardio. 2024 Feb 16;8:e51439. doi: 10.2196/51439 (PMC10907934; doi:10.2196/51439)
Supplement: Multimedia Appendix 9 [file cardio_v8i1e51439_app9.docx]

**Multimedia Appendix 9**

Positive and negative categories among patients with high global satisfaction (score of >3; N=73).

| Dimension | Positive feedback | | Negative feedback | |
| --- | --- | --- | --- | --- |
|  | Number of participants | Subcategory and example quotes | Number of participants | Subcategory and example quotes |
| Assurance: the knowledge and courtesy of the clinicians and their ability to inspire trust and confidence | 54 | - Effective communication (n=24) - “Practitioner was really great and went over and above.” [R35] - “Since I had the first interview, that was fine and everything was well explained to me.” [R43] - “The nurse practitioner was very informative. I was put at ease.” [R87] - Competent clinician (n=14) - “I think that they were knowledgeable of the medications I was taking.” [R31] - “Was very professional.” [R40] - “Competent.” [R39] - Appropriate to their situations (n=11) - “I think all the clinical activities that need to be done have been done. I’ve been perfectly aware of the medications and feel quite fine.” [R31] - Trust (n=5) - “In good hands.” [R15 | 58 | - Incomplete clinical components (n=28) - “If physical exams are necessary, they should be done in person.” [R35] - Incomplete clinical components (n=28) - “If physical exams are necessary, they should be done in person.” [R35] - “Explaining diagnoses, or adjusting medications that can be complex.” [R62] - “Sometimes doctors can spot something that he or she won’t see during phone calls.” [R15] - “An initial consultation would be best in person.” [R55] - Inadequate communication (n=29) - “Sometimes I don’t feel like I can explain or take the time.” [R15] - “There is much to gain in a face-to-face situation. Body language, additional prompts, etc., are helpful in person.” [R86] - “The practitioner had an accent that I found a little challenging to understand all her words on a cell phone call.” [R50] - “Also email the doctor’s advi(s)ce as it is on the phone and one can lose some attention.” [R6] - “Explaining diagnoses, or adjusting medications that can be complex.” [R62] - Lack of rapport and confidence (n=2) - “You have more rapport: facial expressions, personal contact as opposed to a voice [R18] |
| Empathy: caring and understanding, which a company provides or offers its customers in terms of its individualized and personalized attention | 34 | - Friendly (n=14), attentive (n=4), care (n=5), helpful (n=4), good listener (n=4), patient (n=4), and ease (n=3)   - “Pleasant and friendly manner.” [R69]   - “Attentive.” [R31]   - “Showed that they cared.” [R37]   - “They are sincere and helpful at all times.” [R61]   - “Let me express myself without interrupting.” [R39]   - “F was very nervous before the meeting, but once he meet her, she put him at ease.” [R84] | 11 | - Lack of personal connection (n=11)   - “It’s the personal contact that I like. Being an older person, I think we need that, because we’ve always had that in the past.” [R44]   - “Distant.” [R48] |
| Responsiveness: willingness to provide help and a prompt service to customers | 29 | - Convenience (n=18)   - “The more that can be done over the phone, the better. It saves time, travel, energy.” [R6] - Prompt consult (n=10)   - “Much faster and [convenient in the comfort of my own home].” [R28] - Accommodating (n=1)   - “Accommodating.” [R24] | 7 | - Slow response and difficulty reaching the clinic (n=7) - “When you leave a message, I know that they’re not going to get back to you immediately, but should at least acknowledge.” [R37] |
| Reliability: ability to perform the promised service responsibly and accurately | 11 | - Efficient (11)   - “They are right on the ball.” [R61] | 14 | - Administrative issues—lack of preparation (n=9)   - “I don’t know what the purpose of the appointment, what the telecommunications service is going to be about.” [R43]   - “I had been told when I went to the stroke clinic that I was going to be seeing a Dr. M.” [R43]   - “Perhaps they could communicate better with the cardiac doctor before phoning me.” [R53] - During the visit—short and rushed (n=2)   - “It feels a bit rushed.” [R80] |
| Tangibles: technical and home environment experiences | 5 | - No technical problems (n=3)   - “No technical problems as it was a phone call.” [R89] - Home comfort (n=2)   - “It’s better for me to be at home.” [R23] | 8 | - Adding video (n=3)   - “The only thing I would suggest is maybe using Zoom, but I found them easy to talk to.” [R88] - Connectivity issues (n=3)   - “I am in the tech field, and even I had issues connecting to the video call quickly.” [R6] - Poor quality of the voice (n=1)   - “The SPC^a^ staff had echo in her phone.” [R54] - Lack of confidence in technology (n=1)   - “Younger people are more computer-savvy, and can express themselves over the phone or video more easily.” [R44] |

^a^SPC: Stroke Prevention Clinic
